# Dynamics of Progesterone, TNF-***α***, and a Metabolite of PGF2***α*** in Blood Plasma of Beef Cows following Embryo Transfer

**DOI:** 10.1155/2014/650272

**Published:** 2014-10-02

**Authors:** M. C. Mason, J. Copeland, E. J. Cuadra, T. H. Elsasser, Y. Jung, J. Larson

**Affiliations:** ^1^Department of Agriculture, Alcorn State University, 1000 ASU Drive No. 750, Alcorn State, Lorman, MS 39096, USA; ^2^Bovine Functional Genomics Laboratory Beltsville Agricultural Center, USDA ARS, Beltsville, MD 20705, USA; ^3^Cooperative Agricultural Research Center, Prairie View A&M University, P.O. Box 519, AGRL No. 112, Prairie View, TX, USA; ^4^Department of Animal and Dairy Sciences, Mississippi State University, Mississippi State, MS 39762, USA

## Abstract

Lactating beef cows previously synchronized for estrus (d 0) were assigned to four treatments to assess their effectiveness in increasing blood progesterone (P4) and its effects on tumor necrosis factor-*α* (TNF-*α*) and prostaglandin F2*α* (PGF2*α*) after the transfer of embryos. At the time of transfer (d 7), cows received no treatment (control; *n* = 16), a controlled internal drug releasing device (CIDR; *n* = 16), human chorionic gonadotropin (hCG; *n* = 15), or gonadotropin releasing hormone (GnRH; *n* = 15). Blood samples were taken on d 7, 14, and 21 for analysis of P4 and tumor necrosis factor-*α* (TNF-*α*). Blood was collected (every 15 min for 2 h) in half the animals in each treatment group on d 14 and the remaining half on d 21 for analysis of prostaglandin F2*α* metabolite (PGFM). Retention rates were 56.2, 62.5, 46.7, and 13.3% for cows in the control, CIDR, hCG, and GnRH groups, respectively. Progesterone was greater (*P* ≤ 0.05) in cows receiving hCG compared to others on d 14. Progesterone in all treatment groups increased from d 7 to d 14 and declined (*P* ≤ 0.05) from d 14 to d 21. Contrary to pregnant cows, P4 and TNF-*α* declined from d 7 to d 21 in nonpregnant cows (*P* ≤ 0.05). Although PGFM increased by d 21, there was no difference between pregnant and nonpregnant cows.

## 1. Introduction

Progesterone (P4) is abundantly reported in the literature as the primary and most intrinsic hormone associated with embryonic survival during early pregnancy [1, 2]. The emphasis in studying P4 is due to the ability exhibited by P4 in regulating uterine receptivity during implantation [3]. Moreover, low concentrations of P4 are associated with retention of pregnancy in beef cows [4]. Therefore, several studies have been recently designed to examine the effects of supplementing exogenous P4 on embryonic retention after the transfer of embryos. At present, this is commonly done by inserting a controlled internal drug releasing device (CIDR) at the time of breeding.

Our laboratory previously reported [5] that inserting a CIDR immediately after the transfer of embryos enhanced retention rates in recipient lactating and nonlactating beef cows. Similar results have been observed in cattle by other investigators [6, 7]. Conversely, Purcell et al. [5] did not detect beneficial effects on pregnancy rates by placing CIDR's immediately subsequent to embryo transfer in dairy cows.

Several factors might be responsible for the inconsistency in the research outcomes observed by supplementing exogenous P4 aimed to enhance embryonic retention of transferred embryos. In some cases, this may be attributed to the fact that a single CIDR may not deliver enough P4 to support pregnancy on recipients experiencing low circulating P4 [8] or perhaps failing to supplement adequate levels of P4 [6] at the time when majority of embryonic rejections have been suggested to occur after the transfer [7, 9, 10]. Furthermore, several trials have clearly demonstrated that exogenous supplementation of P4 impairs endogenous luteal production of P4 [10, 11] and caused marked regression of the corpus luteum (CL) during early pregnancy in cattle [12]. A more recent study conducted by our laboratory showed cows in the control group having increased pregnancy rates in parallel with increased concentrations of P4 during the first week after embryo transfer compared to a treated group with exogenous P4 via CIDR's [10]. Therefore, strategies to enhance endogenous production of P4 may be an alternative method to examine its role on key factors associated with embryonic retention of transferred embryos.

Some of the potential strategies to achieve this goal may be by either inducing the formation of an accessory CL or by boosting the synthesis of luteal tissue in the existing CL with the use of hormones. Hence, several hormonal treatments have been reported to manipulate secretion of endogenous P4 in cattle. Gonadotropin releasing hormone (GnRH) is reported to alter the synthesis of P4 [13] by manipulating growth of the follicle [14] and number of CL [15]. Consequently, administration of GnRH at the time of insemination results in increased conception rates in cattle [16–18]. In addition, human chorionic gonadotropin (hCG) has also commonly been used in cattle to boost endogenous concentrations of P4 in blood [19–21]. The increase in P4 may be a result of the formation of an accessory CL [22, 23] combined with promoting growth of the existing CL [22, 24]. Nevertheless, hCG inconsistently improves pregnancy rates [23–25]. Consequently, these hormones (GnRH and hCG) were used to boost endogenous P4 in the present study.

Prostaglandin F2*α* (PGF2*α*) and tumor necrosis factor-alpha (TNF-*α*) have been linked to retention of pregnancy by several investigators. Thus, it is well known that PGF2*α* is responsible for inducing regression of the CL [26], which is synthesized by the uterus and regulated by P4 [27]. In cyclic sheep, loss of P4 receptors allows for the uterine release of luteolytic pulses of PGF2*α* suggesting an inverse relationship. Tumor necrosis factor-alpha has both luteotropic and luteolytic functions [28]. Progesterone is considered to be a potent inhibitor of TNF-*α* messenger RNA (mRNA) and TNF-*α* protein production [29]. A decrease in TNF-*α* concentration on d 7 after the transfer of embryos may be associated with the decreased concentrations of P4 observed in the nonpregnant animals in a previous trial [10]. Therefore, the objective of this study was to assess the effectiveness of four treatments in increasing blood P4 and its effects on TNF-*α* and PGF2*α*. Our working hypothesis was that high concentrations of circulating P4 creates a window of time that facilitates synchrony between the embryo and the uterine environment by regulating concentrations of PGF2*α* and TNF-*α* in the uterine environment of the recipient.

## 2. Materials and Methods

### 2.1. Experimental Design and Hormonal Protocol

The study was approved by the Institutional Animal Care and Use Committee of  Mississippi  State University (11-023) and implemented at The Coastal Plain Branch Experiment Station of Mississippi State University in Newton, MS in the Spring of 2011. Lactating Angus crossbred cows were synchronized for estrous by receiving a CIDR (Eazi-Breed CIDR; Zoetis, Madison NJ) for 7 d. One d after removal, all cows (*n* = 62) received an injection of PGF2*α* (25 mg IM; Lutalyse; Zoetis). Cows were observed for estrus (d 0) four times per d (1 h at each time) during the 80 h post-PGF2*α*. Following manual evaluation of the CL via palpation per rectum, all cows exhibiting estrus with a CL received an embryo in the uterine horn ipsilateral to the CL on 7 d after estrus. At the time of transfer, cows were assigned to 1 of 4 treatments: no further treatment (Control, *n* = 16), a CIDR insert (CIDR, *n* = 16), an injection of hCG (1000 IU, IM; Sioux Biochemical, Inc, Sioux Center, IA; hCG, *n* = 15) or an injection of GnRH (100 *μ*g, IM; Cystorelin; Merial, Duluth, GA; GnRH, *n* = 15).

### 2.2. Animals and Embryos

Animals were body condition scored (scale of 1 = emaciated; 9 = obese) by visual appraisal at the beginning of the project according to Whitman [30]. Embryos used in the study were donated by Mississippi State University. Flushing and freezing of the embryos were performed on d 7 after insemination. Embryos were a quality grade 1 [31] and developmental stages 4 and 5; the embryos were frozen in ethylene glycol and stored in liquid nitrogen until their use. The transfer of embryos was performed by an embryo transfer practitioner (Mid-South Reproductive Services, Baton Rouge, LA). Pregnancy diagnosis via palpation per rectum was determined at 60 d after transfer of the embryos.

### 2.3. Collection and Laboratory Analysis of Blood Samples

All samples were collected in 6.0 mL plastic vacutainers with no additives (Fisher Scientific, Pittsburg, PA) from the tail vein. Immediately after collection the samples were stored on ice until they could be centrifuged for 15 min at 1800 g, which was followed by long term storage at −20°C until later analysis. Blood samples for determination of 13, 14-dihydro-15-keto PGF2*α* metabolite (PGFM) were collected from half the animals within each treatment group on d 14 and the remaining half on d 21. On each of these two days, animals selected for collection of blood were additionally divided in two groups and collected every 15 min for 2 h in two individual restraining systems. Synthesis of PGF2*α* in each blood sample was inhibited as previously described by [32]. Blood samples were collected from all cows on d 7 (day of transfer), d 14, and d 21 for analysis of P4 and TNF-*α*.

The concentration of P4 in peripheral blood plasma was determined via radioimmunoassay that has been validated for use in bovine (Coat-a-Count Progesterone, Los Angeles, CA) and used according to the manufacturer's procedure. Plasma samples were assayed for concentrations of  TNF-*α* via a double antibody radioimmunoassay as described by Kenison et al. [33], with the following changes. Antibody (rabbit anti-bovine TNF-*α* R7-93) generated against recombinant bovine TNF-*α* (kindly donated by Ciba-Geigy, Basel, Switzerland) was used as the primary antibody at a final tube dilution of 1 : 120,000 and recombinant bovine TNF-*α* (Kingfisher Biotech, St. Paul, MN) was radioiodinated and used as the assay tracer. Concentrations of PGFM were measured using an enzyme-linked immunosorbent assay (ELISA; Oxford Biomedical Research, Oxford, MI) and used according to the manufacturer's instructions. The intraassay and interassay coefficients of variation were 6.25 and 9.38%, respectively.

### 2.4. Statistical Analysis

Body condition scores of experimental animals were analyzed using the GLM procedure (SAS, Inst. Inc., Cary, NC). Data on conception rates (%) was also analyzed using the GLM procedure with a significance level of 5%; treatment means were compared using the Duncan multiple range test. Concentrations of P4, TNF-*α*, and PGFM in blood were analyzed using the MIXED procedure SAS (SAS Inst., Inc.) with repeated measures. The repeated measures model for the response plasma hormone concentrations on d 0, d 7, and d 14 contained the fixed effect of the treatments and the repeated factors of day and their corresponding interactions. Least squares means by the Bonferroni adjustment were analyzed and separated when a protected *F* test of *P* ≤ 0.05 was detected. Correlation between P4 and PGFM concentrations were performed using the CORR procedure of SAS (SAS Inst., Inc.). All comparisons in the statistical analysis were established at a 5% level of significance. Throughout results, LSMeans ± standard errors are presented.

## 3. Results and Discussion

### 3.1. Progesterone

It is well documented in the literature that body condition of animals influences systemic P4 concentrations of cows [34, 35]. No significant differences were observed in body condition scores among cows in the hCG (5.76 ± 0.21), control (5.47 ± 0.18), GnRH (5.68 ± 0.11), and CIDR (5.67 ± 0.49) groups of this study. Pregnancy diagnosis via palpation per rectum at 60 d after transfer of the embryos revealed retention rates of 56.2% (9/16) for the control group, 62.5% (10/16) for the CIDR group, 13.3% (2/15) for the GnRH group, and 46.6% (7/15) for the hCG group. Pregnancy rates were not different between cows in the control, CIDR, and hCG groups (*P* > 0.05); however, percent pregnancy rate was lower (*P* < 0.05) in the GnRH group when compared to the control and CIDR groups. Other investigators have also observed a negative effect on conception rates in lactating dairy cows receiving treatment with GnRH right after artificial insemination [36]. Nevertheless, it has been shown to improve conception rate in repeat-breeder dairy cows when injected at the time of the fourth insemination [37].

**Figure 1:**
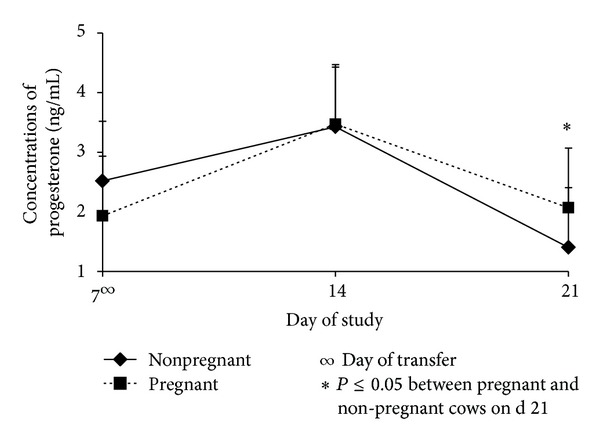
Concentrations (Mean ± SEM) of progesterone in pregnant and nonpregnant cows on d 7, d 14, and d 21.

An overall comparison between pregnant and nonpregnant animals (Figure 1) revealed that pregnant cows had increased (*P* ≤ 0.05) concentrations of P4 on d 21 compared to nonpregnant cows in this study (Figure 1). These results are supported by previous reports revealing that majority of embryo losses occur between d 14 and d 21 of the gestation [7, 9, 10]. However, both nonpregnant and pregnant cows had an increase (*P* ≤ 0.05) in concentration of P4 from d 7 to d 14, but a decrease (*P* ≤ 0.05) from d 14 to d 21. However, regardless of the treatment only nonpregnant cows experienced a significant decrease in P4 (*P* ≤ 0.05) from d 7 to d 21 of this study; this is attributed to the regression of the CL [38] due to factors impairing luteal activity taking place perhaps during the first days after the transfer [7]. Additionally, a previous study revealed that nonpregnant animals bearing a CIDR experienced an increase on P4 from d 7 to d 14 due to a P4 output by the regressing CL combined with the P4 released by the CIDR [10].

There was a significant treatment by pregnancy status interaction with cows failing to maintain pregnancy in the hCG group having significantly greater concentrations of P4 (*P* ≤ 0.05) on d 14 (5.40 ± 0.58 ng/mL) and d 21 (2.91 ± 0.61 ng/mL) compared to nonpregnant cows in any other treatment groups on d 14 (2.27 ± 0.63, 2.32 ± 0.68, and 2.57 ± 0.44 ng/mL) and on d 21 (0.91 ± 0.63, 0.46 ± 0.44, and 1.24 ± 0.47 ng/mL) for the control, CIDR, and GnRH groups, respectively. Although nonpregnant cows in the control and CIDR groups had similar concentrations on d 7 and d 14, a decrease (*P* ≤ 0.05) in the concentration of P4 occurred from d 14 (2.28 ± 0.56, 2.31 ± 0.68 ng/mL) to d 21 (0.90 ± 0.56, 0.46 ± 0.68 ng/mL; Table 1); Animals in these same two experimental groups are the only groups in the study experiencing a decrease in P4 from d 7 to d 21. Non-pregnant animals in the GnRH group also had a decline (*P* ≤ 0.05) in P4 from d 14 (3.34 ± 0.44 ng/mL) to d 21 (1.24 ± 0.47 ng/mL); whereas, animals in the hCG group had an increase (*P* ≤ 0.05) from d 7 (2.67 ± 0.59 ng/mL) to d 14 (5.4 ± 0.59 ng/mL); nevertheless, they similarly had a decrease (*P* ≤ 0.05) in concentration of P4 from d 14 (5.4 ± 0.59 ng/mL) to d 21 (2.90 ± 0.62 ng/mL). Conversely, animals that maintained pregnancy in the control, CIDR and hCG group had an increase (*P* ≤ 0.05; Table 1) in P4 from d 7 (2.27 ± 0.49, 1.54 ± 0.33, 2.17 ± 0.49 ng/mL) to d 14 (3.44 ± 0.49, 2.98 ± 0.36, 4.53 ± 0.78 ng/mL) along with a significant decline from d 14 to d 21.

It is believed that hCG may have increased overall secretion of P4 from the primary CL as well as from an induced secondary luteal structure during the first week of the study [39, 40]. Moreover, Mason et al. [10] also observed a significant increase in P4 7 d after the transfer in control and CIDR-treated cows retaining the embryos to completion of pregnancy.

**Figure 2:**
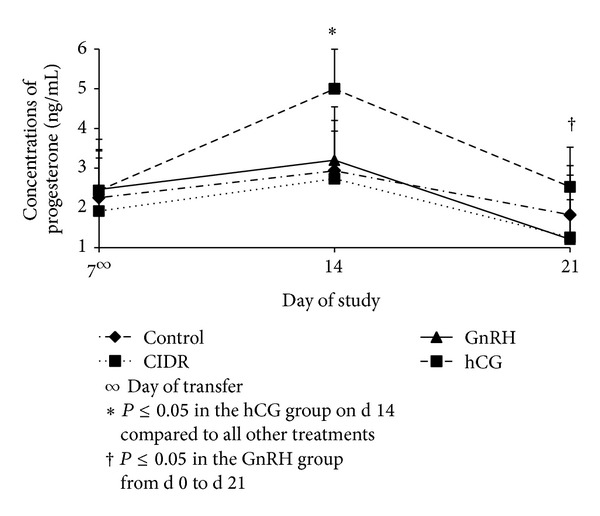
Concentrations (Mean ± SEM) of progesterone among treatment groups on d 7, d 14, and d 21. Treatment by day, concentration of progesterone decreased in cows from all treatments from d 14 to d 21 (*P* ≤ 0.05); CIDR = controlled drug release; GnRH = gonadotropin releasing hormone; hCG = human chorionic gonadotropin.

Concentrations of P4 between treatment groups were not different at the time of transfer of the embryos (Figure 2) as a result of the previously synchronized estrus and the examination of the viability and presence of a well-developed CL in all animals on that day. Concentrations of P4 decreased (*P* ≤ 0.05) from d 14 to d 21 in cows from all treatment groups; however, only cows within the GnRH group experienced decline in P4 concentrations (*P* ≤ 0.05) from d 7 to d 21. This is in line with previous reports indicating that GnRH directly downregulates P4 release [41, 42]. On d 14, cows in the hCG group had increased concentrations of P4 compared to animals in all other treatment groups. On d 21, concentrations of P4 in cows in the hCG group were only greater (*P* ≤ 0.05) than those in the GnRH group on that same day. Also, cows in the hCG group were the only ones with an increase (*P* ≤ 0.05) in P4 from d 7 to d 14.

### 3.2. Tumor Necrosis Factor-*α*

**Figure 3:**
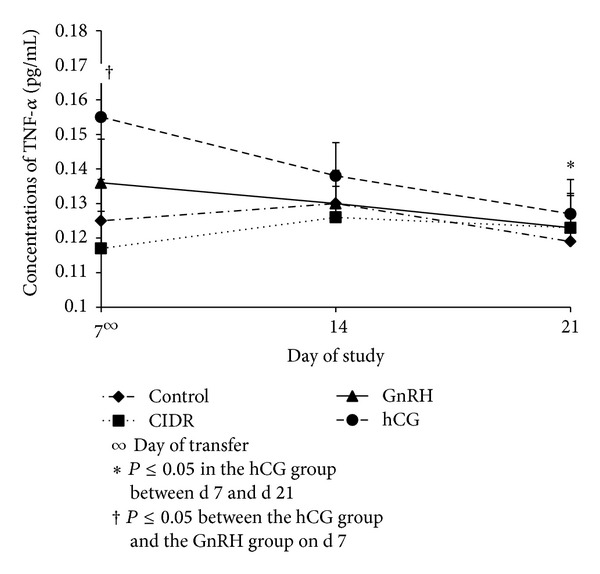
Concentrations (Mean ± SEM) of TNF-*α* among treatment groups on d 7, d 14, and d 21. Treatment *x* day interaction (*P* ≤ 0.05); CIDR = controlled internal drug release; GnRH = gonadotropin releasing hormone; hCG = human chorionic gonadotropin.

Concentrations of TNF-*α* declined (*P* ≤ 0.05) in animals in the hCG group from d 7 to d 21 (Figure 3). This same figure also shows a greater (*P* ≤ 0.05) concentration of TNF-*α* in the hCG group compared to the GnRH group on d 7. The decrease (*P* ≤ 0.05) in TNF-*α* between d 14 and d 21 also follows the decrease (*P* ≤ 0.05) in concentrations of P4 within the hCG group.

The similar pattern of concentration between P4 and TNF-*α* suggests some type of link that allows this hormone and protein to act congruently [43]. When the treatment groups were looked at individually between the pregnant and nonpregnant cows (Table 2), decreased (*P* ≤ 0.05) concentrations of TNF-*α* from d 7 to d 21 were observed in the nonpregnant cows of the hCG group; additionally, an increased (*P* ≤ 0.05) concentration of TNF-*α* in the pregnant cows was observed when compared to the nonpregnant cows on d 21 on that same group. Similar results were observed in a previous study where concentrations of TNF-*α* increased after hCG administration, suggesting a relationship between hCG and TNF-*α* via the Interleukin-6 receptor system [44, 45].

**Figure 4:**
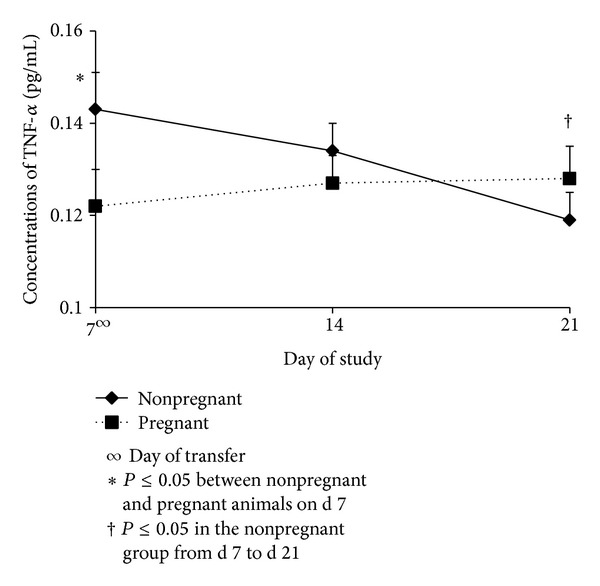
Concentrations (Mean ± SEM) of TNF-*α* in pregnant and nonpregnant cows on d 7, d 14, and d 21. Pregnancy status *x* day interaction (*P* ≤ 0.05); CIDR = controlled internal drug release; GnRH = gonadotropin releasing hormone; hCG = human chorionic gonadotropin.

An overall comparison between pregnant and nonpregnant animals (Figure 4) showed a decrease (*P* ≤ 0.05) in concentration of TNF-*α* from d 7 to d 21 in nonpregnant cows, which occurred similarly in P4 in this same experimental group. Previously, it has been reported by our laboratory that low concentrations of  TNF-*α*  are linked to low concentrations of P4 in nonpregnant cows [10]. The nonpregnant group additionally showed a greater (*P* ≤ 0.05) concentration of TNF-*α* on d 7 compared to the pregnant group for reasons unable to be determined with these results. However, it is noteworthy that contrary to nonpregnant animals, pregnant cows maintained steadier concentrations of TNF-*α* through the entire experimental period. Interestingly, TNF-*α* has been reported having luteolytic properties. Some investigators [46–48] have suggested that TNF-*α* is deleterious to young embryos and promotes the process of luteolysis, thereby stimulating the release of PGF2*α*. On the other hand, other investigators [28] suggest that TNF-*α* may provide both luteolytic and luteotropic tendencies. Thus, the increased concenrations of P4 in pregnant animals may have played a role in inhibiting the luteolytic properties of TNF-*α* [49, 50]; nevertheless, the decreasing concentrations of TNF-*α* in the nonpregnant cows, seems to be associated with the luteolytic properties and consequently low concentrations of P4 as it has been reported in some other species and in cattle [28, 51].

### 3.3. Prostaglandin F2*α*

There were no significant differences in concentrations of PGFM (*P* ≥ 0.05) between the treatment groups on d 14 (0.30 ± 0.48, 0.37 ± 0.84, 0.33 ± 0.46, 0.43 ± 0.99 pg/mL in control, CIDR, GnRH, and hCG groups, resp.) or d 21 (0.51 ± 0.11, 0.64 ± 0.69, 0.55 ± 0.14, 0.62 ± 0.85 pg/mL in control, CIDR, GnRH, and hCG groups, resp.) or between the pregnant and nonpregnant animals within treatment groups. Many studies have associated increased concentrations of PGF2*α* with the termination of pregnancy [26], as PGF2*α* is released from the uterus to essentially cause spontaneous luteolysis in cattle. However, in these current data, animals in the pregnant group actually had more steady concentrations of PGF2*α* on d 21, inferred from the measurement of PGFM, compared to the nonpregnant group on that same day (Table 3).

Prostaglandin F2*α* is released in pulses from the endometrium of the uterus and 80% of it is metabolized during one passage of the lungs, which helps create a short half-life for PGF2*α* as well as fluctuations in concentrations [52]. As expected, variation existed among the six samples collected over the 2 h period for each cow. These data are supported by fellow investigators [32, 53] who also reported variations within concentration of PGF2*α* between cyclic and noncyclic ewes. With the exception of pregnant cows on d 21, on both d 14 and d 21 there were consistently one or two samples within both the pregnant and nonpregnant animals that were different (*P* ≤ 0.05) than the other samples collected on one of those days (Table 3). Furthermore, concentrations of PGFM in samples 5 and 6 within d 21 were significantly increased (*P* ≤ 0.05) in the pregnant (0.69 ± 0.69; 0.57 ± 0.69) cows compared to the nonpregnant cows (0.45 ± 0.49; 0.40 ± 0.49), respectively. Nevertheless, concentrations of PGFM were not correlated on either d 14 or d 21 of the study with concentrations of P4. These findings are supported by other investigators [27, 54] who found that PGF2*α* actually increases during pregnancy. This suggests that the pattern of uterine secretion is altered during pregnancy and that this increased concentration of PGF2*α* now becomes luteo-protective rather than a luteolytic pattern of secretion [55]. One possible luteo-protective mechanism for the pregnant animal is to lower their sensitivity to the luteolytic effects of PGF2*α* [56]. Sensitivity may be lowered by the steady release of PGF2*α* in pregnant animals, where nonpregnant animals have more peaks and variations in their PGF2*α* release [57]. This steady release would allow the CL to become desensitized and have less PGF2*α* receptors, which would induce a more rapid metabolism of PGF2*α* to the inactive PGFM. Alternatively, along with the steady secretion of PGF2*α*, the uterus may receive signals by the conceptus via interferon tau to induce the release of PGF2*α*, which would consequently reduce the luteolytic effects of PGF2*α* [58].

## 4. Conclusion

These results indicate that the strategy of boosting endogenous P4 in cattle by injecting GnRH immediately at the transfer of embryos results in low pregnancy rates. Although treatment with hCG resulted in being the best treatment to boost systemic P4, this did not translate into a higher percent pregnancy compared to the other treatments in this study. Instead, similar concentrations of P4 between d 7 and d 21 are more suggestive of the survival of transferred embryos. Furthermore, with the exception of GnRH, pregnant animals in the other experimental groups had a significant increase in concentrations of P4 from d 0 to d 7. In addition, increased concentrations of P4 seem to be linked with TNF-*α*, perhaps by inhibiting the luteolytic effects of TNF-*α* as more of these cows maintained pregnancy. Concentrations of PGFM were steadier in pregnant animals.

## Figures and Tables

**Table 1:** LSMeans and standard errors for concentrations of progesterone (ng/mL) in nonpregnant and pregnant cows within treatments.

Treatment								
Day	Control		CIDR^1^		GnRH^2^		hCG^3^	
	Nonpregnant	Pregnant	Nonpregnant	Pregnant	Nonpregnant	Pregnant	Nonpregnant	Pregnant
7^4^	2.22 ± 0.56^a^	2.27 ± 0.49^a^	2.54 ± 0.42^a^	1.54 ± 0.33^a^	2.57 ± 0.44^a,b^	1.07 ± 1.66^a^	2.67 ± 0.46^a^	2.17 ± 0.49^a^
14	2.28 ± 0.56^a^	3.44 ± 0.49^b^	2.31 ± 0.47^a^	2.98 ± 0.36^b^	3.35 ± 0.44^a^	1.16 ± 1.66^a^	5.40 ± 0.73^b^	4.53 ± 0.78^b^
21	0.90 ± 0.56^b,x^	2.54 ± 0.49^a,y^	0.46 ± 0.33^b,x^	1.73 ± 0.25^a,y^	1.25 ± 0.47^b,x^	0.85 ± 1.66^a,x^	2.94 ± 0.94^a,x^	2.11 ± 0.95^a,x^

^a,b,c^Means within the same column lacking a common superscript are significantly different (*P* ≤ 0.05).

^x,y^Means within row and within treatment group lacking a common superscript are significantly different (*P* ≤ 0.05).

^1^Controlled internal drug release.

^2^Gonadotropin releasing hormone.

^3^Human chorionic gonadotropin.

^4^Day of embryo transfer.

**Table 2:** LSMeans and standard errors for concentrations of TNF-*α* (pg/mL) in nonpregnant and pregnant cows within treatments.

Treatment								
Day	Control		CIDR^1^		GnRH^2^		hCG^3^	
	Nonpregnant	Pregnant	Nonpregnant	Pregnant	Nonpregnant	Pregnant	Nonpregnant	Pregnant
7^4^	0.113 ± 0.02^a^	0.124 ± 0.01^a^	0.129 ± 0.03^a^	0.112 ± 0.01^a^	0.146 ± 0.01^a^	0.095 ± 0.04^a^	0.161 ± 0.02^a^	0.160 ± 0.04^a^
14	0.015 ± 0.02^a^	0.119 ± 0.01^a^	0.129 ± 0.01^a^	0.124 ± 0.01^a^	0.132 ± 0.01^a^	0.102 ± 0.05^a^	0.132 ± 0.02^ab^	0.144 ± 0.02^a^
21	0.116 ± 0.01^a^	0.120 ± 0.01^a^	0.126 ± 0.01^a^	0.122 ± 0.01^a^	0.122 ± 0.01^a^	0.14 ± 0.02^a^	0.115 ± 0.01^b,x^	0.148 ± 0.01^a,y^

^a,b,c^Means within the same column lacking a common superscript are significantly different (*P* ≤ 0.05).

^x,y^Means within row and within treatment group lacking a common superscript are significantly different (*P* ≤ 0.05).

^1^Controlled internal drug release.

^2^Gonadotropin releasing hormone.

^3^Human chorionic gonadotropin.

^4^Day of embryo transfer.

**Table 3:** LSMeans and standard errors for concentrations of PGFM (ng/mL) for d 14 and 21 in nonpregnant and pregnant cows.

^ 1^Serum samples	Pregnant		Nonpregnant		All pregnant	All nonpregnant
	Day 14	Day 21	Day 14	Day 21		
1	0.38 ± 0.60^ab^	0.68 ± 0.69^a^	0.37 ± 0.32^ab^	0.67 ± 0.49^a^	0.49 ± 0.15	0.52 ± 0.15
2	0.36 ± 0.54^ab^	0.68 ± 0.69^a^	0.35 ± 0.32^a^	0.66 ± 0.49^a^	0.52 ± 0.16	0.51 ± 0.16
3	0.33 ± 0.60^a^	0.71 ± 0.69^a^	0.33 ± 0.32^a^	0.67 ± 0.49^a^	0.52 ± 0.19	0.50 ± 0.17
4	0.31 ± 0.54^a^	0.77 ± 0.74^a^	0.30 ± 0.32^a^	0.68 ± 0.52^a^	0.54 ± 0.23	0.49 ± 0.19
5	0.31 ± 0.54^a^	0.69 ± 0.69^a^	0.29 ± 0.32^a^	0.45 ± 0.49^b^	0.50 ± 0.19	0.37 ± 0.08
6	0.51 ± 0.54^cb^	0.57 ± 0.69^a^	0.46 ± 0.32^b^	0.40 ± 0.49^b^	0.54 ± 0.03	0.43 ± 0.03

Mean	0.36 ± 0.03^x^	0.68 ± 0.03^y^	0.35 ± 0.03^x^	0.59 ± 0.05^y^	0.52 ± 0.16^x^	0.47 ± 0.12^x^

^a,b,c^Means within the same column lacking a common superscript are significantly different.

^x,y^Means within row and within treatment group lacking a common superscript are significantly different (*P* ≤ 0.05).

(*P* ≤ 0.05).

^1^Serum samples taken 15 minute apart on each day.

## References

[1] Parkinson TJ, Lamming GE (1990). Interrelationships between progesterone, 13,14-dihydro-15-keto PGF-2*α* (PGFM) and LH in cyclic and early pregnant cows. *Journal of Reproduction and Fertility*.

[2] Macmillan KL, Taufa VK, Day AM, Peterson AJ (1991). Effect of supplemental progesterone on pregnancy rates in cattle. *Journal of Reproduction and Fertility*.

[3] Cheon Y-P, Xu X, Bagchi MK, Bagchi IC (2003). Immune-responsive gene 1 is a novel target of progesterone receptor and plays a critical role during implantation in the mouse. *Endocrinology*.

[4] Burke JM, De La Sota RL, Risco CA, Staples CR, Schmitt ÉJ-P, Thatcher WW (1996). Evaluation of timed insemination using a Gonadotropin-releasing hormone agonist in lactating dairy cows. *Journal of Dairy Science*.

[5] Purcell SH, Beal WE, Gray KR (2005). Effect of a CIDR insert and flunixin meglumine, administered at the time of embryo transfer, on pregnancy rate and resynchronization of estrus in beef cattle. *Theriogenology*.

[6] van Werven T, Waldeck F, Souza AH, Floch S, Englebienne M (2013). Comparison of two intravaginal progesterone releasing devices (PRID-Delta vs CIDR) in dairy cows: blood progesterone profile and field fertility. *Animal Reproduction Science*.

[7] Butcher RL, Reber JE, Lishman AW (1992). Maintenance of pregnancy in postpartum beef cows that have short-lived corpora lutea.. *Journal of animal science*.

[8] Rhinehart JD, Starbuck-Clemmer MJ, Flores JA (2009). Low peripheral progesterone and late embryonic/early fetal loss in suckled beef and lactating dairy cows. *Theriogenology*.

[9] Lonergan P (2011). Influence of progesterone on oocyte quality and embryo development in cows. *Theriogenology*.

[10] Mason M, Cuadra EJ, Elsasser TH, Lopez J, Yoonsung J (2013). Evaluating the interaction between progesterone, tumor necrosis factor-alpha and cortisol on early loss of transferred embryo in beef cows. *Canadian Journal of Animal Science*.

[11] Loy RG, Zimbelman RG, Casida LE (1960). Effects of injected ovarian hormones on the corpus luteum of the estrual cycle in cattle. *Journal of Animal Science*.

[12] Zimbelman RG, Pope AL, Casida LE (1959). Effect of exogenous progesterone on the corpus luteum of the bred ewe. *Journal of Animal Science*.

[13] Busch DC, Atkins JA, Bader JF (2008). Effect of ovulatory follicle size and expression of estrus on progesterone secretion in beef cows. *Journal of Animal Science*.

[14] Peters MW, Pursley JR (2003). Timing of final GnRH of the Ovsynch protocol affects ovulatory follicle size, subsequent luteal function, and fertility in dairy cows. *Theriogenology*.

[15] Stevenson JS, Tenhouse DE, Portaluppi MA, Lloyd A (2006). Post-AI intervention in lactating dairy cattle. *Journal of Animal Science*.

[16] Nakao T, Narita S, Tanaka K (1983). Improvement of first-service pregnancy rate in cows with gonadotropin-releasing hormone analog. *Theriogenology*.

[17] Schels HF, Mostafawi D (1978). The effect of Gn-RH on the pregnancy rate of artificially inseminated cows. *The Veterinary Record*.

[18] Stevenson JS, Call EP, Scoby RK, Phatak AP (1990). Double insemination and gonadotropin-releasing hormone treatment of repeat-breeding dairy cattle. *Journal of Dairy Science*.

[19] Beindorff N, Honnens A, Penno Y, Paul V, Bollwein H (2009). Effects of human chorionic gonadotropin on luteal blood flow and progesterone secretion in cows and in vitro-microdialyzed corpora lutea. *Theriogenology*.

[20] de Rensis F, López-Gatius F, García-Ispierto I, Techakumpu M (2010). Clinical use of human chorionic gonadotropin in dairy cows: an update. *Theriogenology*.

[21] Stevenson JS, Portaluppi MA, Tenhouse DE (2007). Interventions after artificial insemination: conception rates, pregnancy survival, and ovarian responses to gonadotropin-releasing hormone, human chorionic gonadotropin, and progesterone. *Journal of Dairy Science*.

[22] Rajamahendran R, Sianangama PC (1992). Effect of human chorionic gonadotrophin on dominant follicles in cows: formation of accessory corpora lutea, progesterone production and pregnancy rates. *Journal of Reproduction and Fertility*.

[23] Santos JEP, Thatcher WW, Pool L, Overton MW (2001). Effect of human chorionic gonadotropin on luteal function and reproductive performance of high-producing lactating Holstein dairy cows. *Journal of Animal Science*.

[24] Marquezini GHL, Dahlen CR, Bird SL, Lamb GC (2011). Administration of human chorionic gonadotropin to suckled beef cows before ovulation synchronization and fixed-time insemination: replacement of gonadotropin-releasing hormone with human chorionic gonadotropin1. *Journal of Animal Science*.

[25] Shams-Esfandabadi N, Shirazi A, Mirshokrai P, Bonyadian M (2007). Influence of hCG administration after AI on conception rates and serum progesterone concentration in cattle. *Pakistan Journal of Biological Sciences*.

[26] Watts TL, Fuquay JW (1985). Response and fertility of dairy heifers following injection with prostaglandin F2*α* during early, middle or late diestrus. *Theriogenology*.

[27] Lewis GS (2003). Role of ovarian progesterone and potential role of prostaglandin F 2*α* and prostaglandin E2 in modulating the uterine response to infectious bacteria in postpartum ewes. *Journal of Animal Science*.

[28] Okuda K, Sakumoto R (2003). Multiple roles of TNF super family members in corpus luteum function. *Reproductive Biology and Endocrinology*.

[29] Miller L, Hunt JS (1998). Regulation of TNF-*α* production in activated mouse macrophages by progesterone. *Journal of Immunology*.

[30] Whitman RW (1975). *Weight change, body condition and beef cow reproduction [Ph.D. dissertation]*.

[31] Putney DJ, Thatcher WW, Drost M, Wright JM, DeLorenzo MA (1988). Influence of environmental temperature on reproductive performance of bovine embryo donors and recipients in the southwest region of the United States. *Theriogenology*.

[32] Pexton JE, Weems CW, Inskeep EK (1975). Prostaglandins F in uterine and ovarian venous plasma from nonpregnant and pregnant ewes collected by cannulation. *Prostaglandins*.

[33] Kenison DC, Elsasser TH, Fayer R (1990). Radioimmunoassay for bovine tumor necrosis factor: concentrations and circulating molecular forms in bovine plasma. *Journal of Immunoassay*.

[34] Vizcarra JA, Wettemann RP, Spitzer JC, Morrison DG (1998). Body condition at parturition an dpostpartum weight gain influence luteal activity and concentrations of glucose, insulin, and noesterified faty acids in plasma of primiparous beef cows. *Journal of Animal Science*.

[35] Selk GE, Wettemann RP, Lusby KS (1988). Relationship among weight change, body condition and reproductive performance of range beef cows. *Journal of Animal Science*.

[36] Shephard RW, Morton JM, Norman ST (2014). Effects of administration of gonadotropin-releasing hormone at artificial insemination on conception rates in dairy cows. *Animal Reproduction Science*.

[37] Phatak AP, Whitmore HL, Brown MD (1986). Effect of gonadotrophin releasing hormone on conception rate in repeat-breeder dairy cows. *Theriogenology*.

[38] Thatcher WW, Hansen PJ, Gross TS, Helmer SD, Plante C, Bazer FW (1989). Antiluteolytic effects of bovine trophoblast protein-1. *Journal of Reproduction and Fertility. Supplement*.

[39] Diaz T, Schmitt EJ-P, De La Sota RL, Thatcher M-J, Thatcher WW (1998). Human Chorionic Gonadotropin-induced alterations in ovarian follicular dynamics during the estrous cycle of heifers. *Journal of Animal Science*.

[40] Fricke PM, Reynolds LP, Redmer DA (1993). Effect of human chorionic gonadotropin administered early in the estrous cycle on ovulation and subsequent luteal function in cows.. *Journal of animal science*.

[41] Harper R, Bennett WA, Cuadra EJ, Vaughn CF, Whitworth NS (2008). Effects of GnRH in combination with PGF2*α* on the dynamics of follicular and luteal cells in post-pubertal Holstein heifers. *Journal of Livestock Science*.

[42] Zerani M, Catone G, Maranesi M, Gobbetti A, Boiti C, Parillo F (2012). Gonadotropin-releasing hormone 1 directly affects corpora lutea lifespan in mediterranean buffalo (*Bubalus bubalis*) during diestrus: presence and in vitro effects on enzymatic and hormonal activities. *Biology of Reproduction*.

[43] Nilsson EE, Stanfield J, Skinner MK (2006). Interactions between progesterone and tumor necrosis factor-*α* in the regulation of primordial follicle assembly. *Reproduction*.

[44] Li Y, Matsuzaki N, Masuhiro K (1992). Trophoblast-derived tumor necrosis factor-*α* induces release of human chorionic gonadotropin using interleukin-6 (IL-6) and IL-6-receptor-dependent system in the normal human trophoblasts. *The Journal of Clinical Endocrinology and Metabolism*.

[45] Montgomery Rice V, Limback SD, Roby KF, Terranova PF (1998). Changes in circulating and ovarian concentrations of bioactive tumour necrosis factor *α* during the first ovulation at puberty in rats and in gonadotrophin-treated immature rats. *Journal of Reproduction and Fertility*.

[46] El-Sayed A, Hoelker M, Rings F (2006). Large-scale transcriptional analysis of bovine embryo biopsies in relation to pregnancy success after transfer to recipients. *Physiological Genomics*.

[47] Hansen PJ, Block J (2004). Towards an embryocentric world: The current and potential uses of embryo technologies in dairy production. *Reproduction, Fertility and Development*.

[48] Niswender GD, Juengel JL, Silva PJ, Rollyson MK, McIntush EW (2000). Mechanisms controlling the function and life span of the corpus luteum. *Physiological Reviews*.

[49] Candolfi M, Jaita G, Zaldivar V (2005). Progesterone antagonizes the permissive action of estradiol on tumor necrosis factor-*α*-induced apoptosis of anterior pituitary cells. *Endocrinology*.

[50] Luo G, Abrahams VM, Tadesse S (2010). Progesterone inhibits basal and tnf-*α*-induced apoptosis in fetal membranes: a novel mechanism to explain progesterone-mediated prevention of preterm birth. *Reproductive Sciences*.

[51] Bilodeau-Goeseels S, Kastelic JP (2003). Factors affecting embryo survival and strategies to reduce embryonic mortality in cattle. *Canadian Journal of Animal Science*.

[52] Nett TM, Staigmiller RB, Akbar AM, Diekman MA, Ellinwood WE, Niswender GD (1976). Secretion of prostaglandin F2alpha in cycling and pregnant ewes. *Journal of Animal Science*.

[53] Thorburn GD, Cox RI, Currie WB, Restall BJ, Schneider W (1973). Prostaglandin F and progesterone concentrations in the utero-ovarian venous plasma of the ewe during the oestrous cycle and early pregnancy. *Journal of Reproduction and Fertility*.

[54] Burgess KM, Ralph MM, Jenkin G, Thorburn GD (1990). Effect of oxytocin and estradiol on uterine prostaglandin release in nonpregnant and early-pregnant ewes. *Biology of Reproduction*.

[55] Choudhary E, Costine BA, Wilson ME, Inskeep EK, Flores JA (2004). Prostaglandin F2*α* (PGF2*α*) independent and dependent regulation of the bovine luteal endothelin system. *Domestic Animal Endocrinology*.

[56] Silvia WJ, Niswender GD (1986). Maintenance of the corpus luteum of early pregnancy in the ewe. IV. Changes in luteal sensitivity to prostaglandin F2*α* throughout early pregnancy. *Journal of Animal Science*.

[57] Ellinwood WE, Nett TM, Niswender GD (1979). Maintenance of the corpus luteum of early pregnancy in the ewe. I. Luteotropic properties of embryonic homogenates. *Biology of Reproduction*.

[58] Niswender GD, Juengel JL, McGuire WJ, Belfiore CJ, Wiltbank MC (1994). Luteal function: the estrous cycle and early pregnancy. *Biology of Reproduction*.

